# Temporal trends in dementias in older adults attributable to high fasting plasma glucose from 1990 to 2021 and forecasted disease burden in 2040 in China and globally

**DOI:** 10.3389/fpubh.2025.1584386

**Published:** 2025-06-18

**Authors:** Pinyuan Dai, Jie Yu, Yongxing Lin, Xiaoyan Zhou, Hao Wang, Weiwei Gong, Jin Pan, Yunqi Guan, Jieming Zhong, Na Li, Zuyun Liu

**Affiliations:** ^1^Zhejiang Provincial Center for Diseases Control and Prevention, Hangzhou, China; ^2^Center for Clinical Big Data and Analytics of the Second Affiliated Hospital, Department of Big Data in Health Science School of Public Health, The Key Laboratory of Intelligent Preventive Medicine of Zhejiang Province, Zhejiang University School of Medicine, Hangzhou, China

**Keywords:** global burden of disease, dementia, risk factors, glucose, diabetes, China

## Abstract

**Introduction:**

The Global Burden of Diseases Study systematically updates the dementia burden attributable to high fasting plasma glucose (HFPG) to investigate the temporal trends of dementia burden and promote comparisons between countries, sexes, and age groups. In this study, we aimed to estimate the disease burden of dementia attributable to HFPG using an age-period-cohort model in adults aged >60 years from 1990 to 2021 and forecast the mortality and disability-adjusted life-years (DALYs) rates in China and globally in 2040.

**Method:**

Data on the mortality and DALYs rates of dementia attributable to HFPG in China and globally were extracted from the Global Burden of Disease Study 2021. An age-period-cohort model was used to estimate the net drift, local drift, fitted longitudinal age-specific rates, and period/cohort relative risks from 1990 to 2021. The Bayesian age-period-cohort model was used to predict future mortality and DALYs rates from 2022 to 2040.

**Results:**

The net drifts showed an overall upward trend in the dementia burden attributable to HFPG in China and globally from 1990 to 2021, with a much slower trend in China. A constantly rising risk for age and birth cohort effects was observed, while period effects presented a globally constantly increasing risk and two inflection points in China, probably due to healthcare reform. The forecasted disease burden by 2040 demonstrated an increasing trend globally and a declining trend in China.

**Conclusion:**

The burden of dementia attributable to HFPG has consistently increased globally over the past 30 years but has gradually declined in China in recent years. China’s strategies for preventing and managing diabetes and dementia may provide valuable insights for other regions. Further targeted policies are required to reduce the burden on females and older adults, particularly to improve their quality of life.

## 1 Introduction

Alzheimer’s disease and other dementias (hereafter referred to as dementia) are the most common causes of neurodegenerative diseases and neurological disorders (1). As reported, dementia accounted for 4.4% (2.4 million) of the total deaths, being the fifth leading cause of death worldwide (2). The disease burden of dementia has been well documented and is heterogeneous across different areas. China and Japan have shown an increase in the age-standardized prevalence of dementia, while some countries including the United States of America (USA), United Kingdom (UK), Sweden, and the Netherlands have reported declines (2). In 2021, China accounted for 26.7% (15.3 million) of the global number of dementia cases (3). Notably, among individuals aged >60 years, the estimated number of dementia cases in China is 9.5 million largely due to population aging, with a prevalence of 5.3% (4). The increase in the disease burden of dementia accentuates the importance of developing precise prevention and intervention strategies for dementia in China and globally, especially for older populations.

Accumulating evidence has shown that over one-third of dementia cases could be delayed or prevented by the management of modifiable risk factors (5, 6). Numerous studies have identified multiple modifiable risk factors associated with increased dementia incidence and disease progression, including metabolic risk factors (e.g., hypertension, type 2 diabetes, hyperlipidemia), smoking, and physical inactivity 12 (7–9). Among these risk factors, high fasting plasma glucose (HFPG) is a well-defined risk factor for dementia as well as a leading global disease risk factor. In 2021, the global number of deaths and disability-adjusted life-years (DALYs) attributed to HFPG was 6.5 million and 172.1 million, accounting for 11.30 and 6.41% of all-cause deaths and DALYs, respectively (10). Additionally, due to population aging, unhealthier dietary patterns, urbanization, and the lower physical activity level and higher prevalence of obesity, the global mean level of glucose is rising and HFPG is becoming more popular (11). Specifically, as reported before, higher glucose levels increased the relative risk of dementia among individuals without and with diabetes (12). Evidence have showed that glucose-related mitochondrial dysfunction, oxidative stress, and toxic effects lead to vascular damage and structural changes in learning-related brain areas, subsequently resulting in cognitive impairment and dementia (13–15).

Therefore, ascertaining the dementia burden attributable to HFPG across different countries, age groups, and sexes is critical for prioritizing actions for the precise and targeted prevention of dementia and HFPG. Previous evidence has shown that the proportion of dementia attributable to HFPG among Chinese females aged ≥40 years has slightly decreased in recent years. Is there a similar decreasing trend in the entire older Chinese population? If so, what are the underlying reasons for this change and is this trend also observed in other regions? Addressing these questions may provide insights into global prevention and control strategies. However, the deaths and DALYs due to dementia attributable to HFPG in older populations in China and globally remain unclear. Moreover, an in-depth analysis of the temporal trends in age, period, and birth cohort effects is still lacking. In addition to quantifying the current HFPG-attributable burden of dementia, forecasts of the death and DALYs rates of dementia may help in planning more precise and cost-effective policies and the allocation of resources.

The Global Burden of Diseases Study (GBD) systematically updates the dementia burden attributable to HFPG in 204 countries and territories, creating opportunities to investigate the temporal trends of dementia burden and promote comparisons between countries, sexes, and age groups. In this study, we estimated HFPG-attributable dementia-related deaths and DALYs using an age-period-cohort (APC) model in individuals aged >60 years in China and globally from 1990 to 2021 and forecasted the death and DALYs rates in 2040. Our study aimed to provide proposals for policy and program implementation to increase awareness of HFPG-attributable dementia and improve the diagnosis and control of HFPG to reduce the dementia burden.

## 2 Materials and methods

### 2.1 Study data

Data on the burden of Alzheimer’s disease and other dementias attributable to HFPG in China and globally were collected using the Global Health Data Exchange GBD 2021 Results Tool (http://ghdx.healthdata.org/gbd-results-tool), which quantifies the disease burden related to risk factors worldwide from 1990 to 2021. This study complied with the Guidelines for Accurate and Transparent Health Estimation Reporting and informed consent was not required.

In the GBD 2021, the burden of nonfatal disease was modeled using Disease Modeling Meta-Regression (DisMod-MR) 2.1, a Bayesian compartmental model (16). Data on risk factors were presented in the population attributable fraction (PAF) of a disease attributable to a particular risk factor with a 95% uncertainty interval (UI). The PAF is the proportion of the cause that will decrease if the exposure to a certain risk factor in the past has been reduced to the theoretical minimum risk exposure level of a certain population (17). The deaths attributed to HFPG were calculated by multiplying the PAF by the number of deaths. Detailed methods for the estimation of dementia burden attributable to HFPG and the general methods of the GBD have been described previously (18, 19).

The dementia burden attributable to HFPG by sex (males, females, and both sexes), country, and eight age categories (5-year groups within the ages of 60–94 years, and ≥95 years) from 1990 to 2021 were also collected. We compared countries based on their sociodemographic indices (SDIs), a composite indicator of health comprising average income per capita, average years of schooling, and total fertility rate (19, 20).

### 2.2 Definitions

The definition of Alzheimer’s disease and other dementia in the GBD 2021 were from the Diagnostic and Statistical Manual of Mental Disorders (DSM) III, III-R, IV, or V, or the International Classification of Diseases (ICD)-8, ICD-9, or ICD-10 (21, 22). The theoretical minimum risk exposure level at which the risk of health outcomes was lowest was 4.5–5.4 mmol/L for HFPG (23).

### 2.3 Measurements

The GBD 2021 estimated the attributable number of deaths, mortality, age-standardized mortality rates (ASMR), years of life lost (YLL, calculated as the number of deaths in each age group multiplied by the remaining life expectancy of that age group), years of life lived with disability (YLD, years lived with health loss weighted by the severity of disability), DALYs, and age-standardized DALYs rates (ASDR) of Alzheimer’s and other dementias attributed to HFPG. DALYs are a comprehensive measure calculated as the sum of YLL and YLD, also defined as the years of healthy life lost, which accounts for premature death and disability (23).

### 2.4 Statistical analysis

The APC model was used to evaluate the contributions of age, period, and cohort to the effect of HFPG on the dementia burden (24, 25). APC analysis can reduce the interaction between the three factors and provide accurate results for each effect. The age effect refers to the effects of changes in biological aging. Period effect refers to changes in mortality and DALY rates over time that simultaneously affect all age groups. This can be attributed to developments in disease classification, screening methods, and medical techniques. The cohort effect represents long-term trends in disease mortality and DALY rates influenced by group lifestyles, exposure to risk factors, and changing environments. In this study, estimates of net drift, local drift, longitudinal age curve, period rate ratio (RR), and cohort RR were used to demonstrate the effects of age, period, and cohort. Net drift (% per year) refers to the overall annual percentage change in the ASMR and ASDR over time. The local drift refers to the annual percentage change of mortality and DALY rates for each age group and represents the log-linear trend by period and birth cohort (26). The longitudinal age curve is the fitted longitudinal age-specific rate adjusted for period bias in the reference cohort. Period RR or cohort RR reflect the period or cohort relative risk adjusted for age and the nonlinear period or cohort effects versus the reference one (27). Based on recommendations from the literature, we set the 2002–2006 period and 1937–1946 birth cohort as the references (28).

The Bayesian APC model was used to predict future age-specific mortality and DALYs rates of Alzheimer’s disease and other dementias attributed to HFPG from 2022 to 2040 (24, 25). Assuming that the three effects are similar in the adjacent time, the Bayesian APC model adopts a second-order random walk to smooth the priors of age, period, and cohort effects to predict posteriori mortality and DALY rates. In this study, the age, period, and cohort effects were assumed to follow a gamma distribution with parameters (1, 0.00005), and the overdispersion parameter was assumed to follow a gamma distribution with parameters (1, 0.005). This approach combines nested Laplace approximations, which show more favorable coverage and precision than other methods, by avoiding any mixing and convergence problems caused by sampling techniques related to the Markov Chain Monte Carlo. version 4.2.0. All analyses were conducted using R version 4.2.0. The Bayesian APC model was implemented by calling the BAPC package within the INLA package.

## 3 Results

### 3.1 Overall trends in dementia disease burden attributable to HFPG in China and globally

Supplementary Figure 1 shows trends in the incidence and prevalence of dementia in China and globally. The data show that the global incidence and prevalence have remained stable with fluctuations, whereas those in China have exhibited an overall upward trend, reaching a peak increase after 2010. Table 1 presents the deaths, DALYs, YLDs, and YLLs for Alzheimer’s disease and other dementias attributable to HFPG in 1990 and 2021. Table 2 displays the percentage change and net drift in the age-standardized rates in China and globally from 1990 to 2021. Overall, dementia’s disease burden attributable to HFPG in older adults are on an upward trend in China and globally. From 1990 to 2021, the number of dementia-related deaths in older adults in China increased by approximately 350.8%, reaching 58838.24 by 2021. The ASMR was 3.64 (95% UI: 0.14, 11.53) per 100000 population in 2021, a 7.7% increase from 1990. Furthermore, the APC model estimated the net drift of ASMR at 0.36% (95% confidence interval [CI]: 0.29–0.44%) and 1.17% (95% CI: 1.14–1.20%) per year in China and globally, respectively. For DALY, the ASDR was 66.72 (95% UI: 3.91, 177.01) per 100000 population in 2021 in China. The APC model estimated the net drift of the ASDR to be 0.52% (95% CI: 0.48–0.56%) and 1.16% (95% CI: 1.14–1.18%) per year in China and globally, respectively. The trends in YLD and YLL are similar to those described above. Overall, China had a much lower upward trend in dementia disease burden attributable to HFPG than at the global level.

**Table 1:** The burden of dementia attributable to high fasting plasma glucose in China and globally.

Measure	Location	Number		Age-standardized rate	
		1990	2021	1990	2021
Deaths	China	13051.62	58838.24	3.38 (0.13, 10.63)	3.64 (0.14, 11.53)
Deaths	Global	71470.52	290032.19	2.64 (0.11, 8.38)	3.73 (0.15, 11.84)
DALYs	China	286765.30	1204039.42	57.10 (3.15, 157.09)	66.72 (3.91, 177.01)
DALYs	Global	1441539.86	5348853.67	47.07 (2.72, 126.46)	66.42 (3.83, 178.85)
YLDs	China	84244.20	411880.82	15.40 (1.35, 34.02)	21.99 (1.97, 48.14)
YLDs	Global	462554.86	1687335.50	14.51 (1.25, 32.07)	20.71 (1.78, 45.43)
YLLs	China	202521.10	792158.60	41.69 (1.63, 135.17)	44.74 (1.77, 139.34)
YLLs	Global	978985.00	3661518.17	32.56 (1.31, 101.98)	45.71 (1.85, 143.50)

DALYs, disability-adjusted life years; YLDs, years lived with disability; YLLs, years of life lost.

**Table 2:** Trends in the burden of dementia attributable to high fasting plasma glucose from 1990 to 2021 in China and globally.

Measure	Location	Percentage change, 1990–2021	Net drift (% per year)
Deaths	China	3.51 (2.77, 4.58)	0.36 (0.29, 0.44)
Deaths	Global	3.06 (2.80, 3.46)	1.17 (1.14, 1.20)
DALYs	China	3.20 (2.61, 3.96)	0.52 (0.48, 0.56)
DALYs	Global	2.71 (2.53, 2.93)	1.16 (1.14, 1.18)
YLDs	China	3.89 (3.58, 4.29)	0.91 (0.86, 0.96)
YLDs	Global	2.65 (2.51, 2.77)	1.16 (1.14, 1.18)
YLLs	China	2.91 (2.25, 3.88)	0.34 (0.31, 0.38)
YLLs	Global	2.74 (2.51, 3.07)	1.16 (1.14, 1.18)

DALYs, disability-adjusted life years; YLDs, years lived with disability; YLLs, years of life lost.

**Figure 1:**
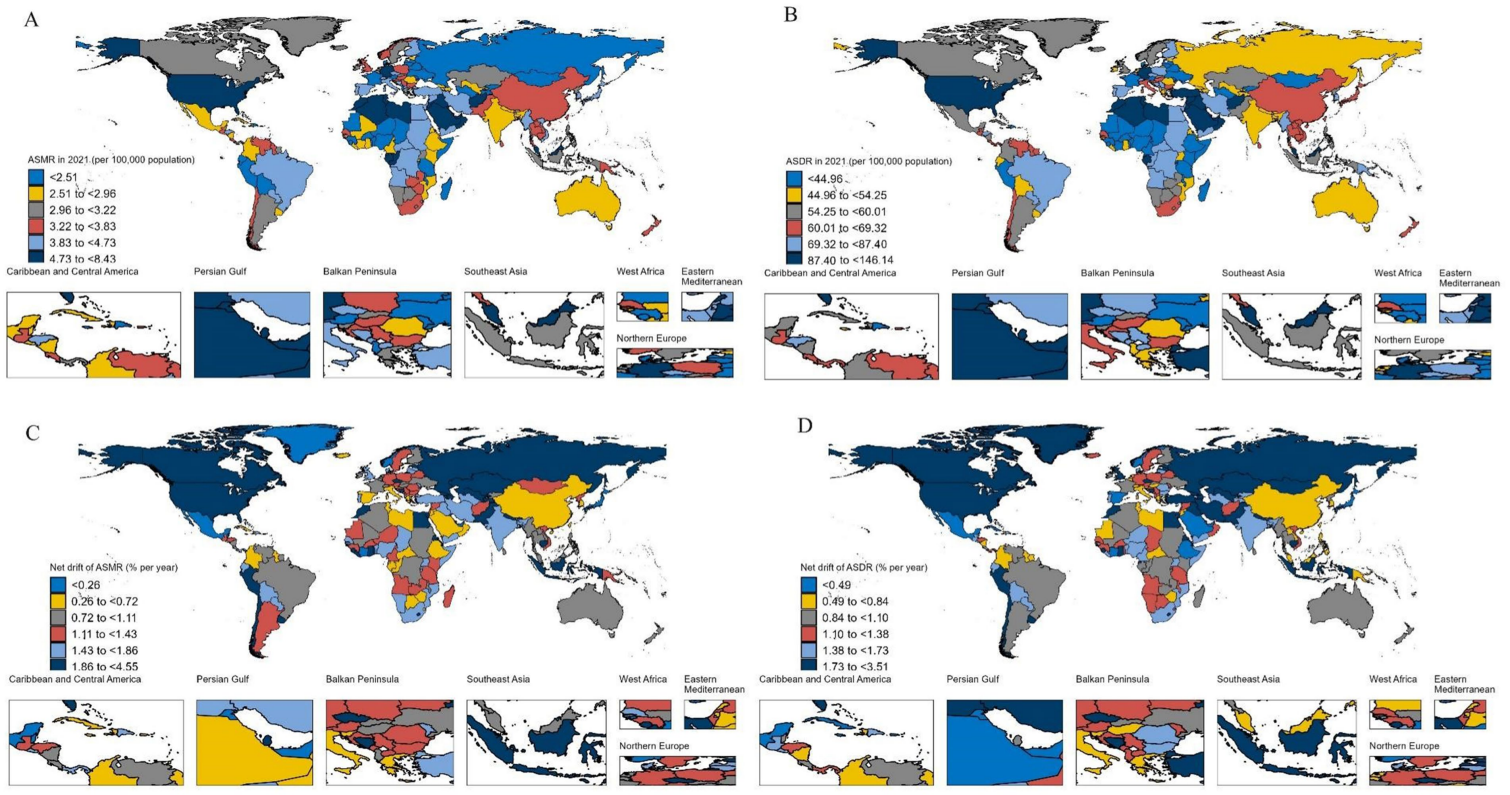
The ASMR and ASDR in 2021 and their net drift from 1990 to 2021 in 204 countries and territories. ASMR, age-standardized mortality rate; ASDR, age-standardized DALY rate; DALYs, disability-adjusted life years. **(A)** ASMR; **(B)** ASDR; **(C)** Net drift of ASMR; **(D)** Net drift of ASDR.

The national ASMR and ASDR in 2021 as well as the net drift of the ASMR and ASDR from 1990 to 2021 are shown in Figure 1 and Supplementary Tables S1, S2. In 2021, among the 204 countries and territories, 35 countries had an ASMR lower than 2.51 (the lower one-sixth), most of which were low-to middle-SDI countries, and 34 countries had an ASMR greater than 4.73 (upper one-sixth), most of which were middle-to high-SDI countries. The average ASMR was 3.48, 3.76, 3.85, 3.47, and 2.93 in the high-, middle-high-, middle-, middle-low-, and low-SDI countries, respectively. Although China had the highest number of Alzheimer’s disease-related deaths attributable to HFPG, owing to its large population, the ASMR was at the upper medium level, with modest net drifts in ASMR. The ASDR and its net drift from 1990 to 2021 were similar to those of the ASMR in China and globally.

### 3.2 Temporal trends in dementia disease burden attributable to HFPG across different age groups

**Figure 2:**
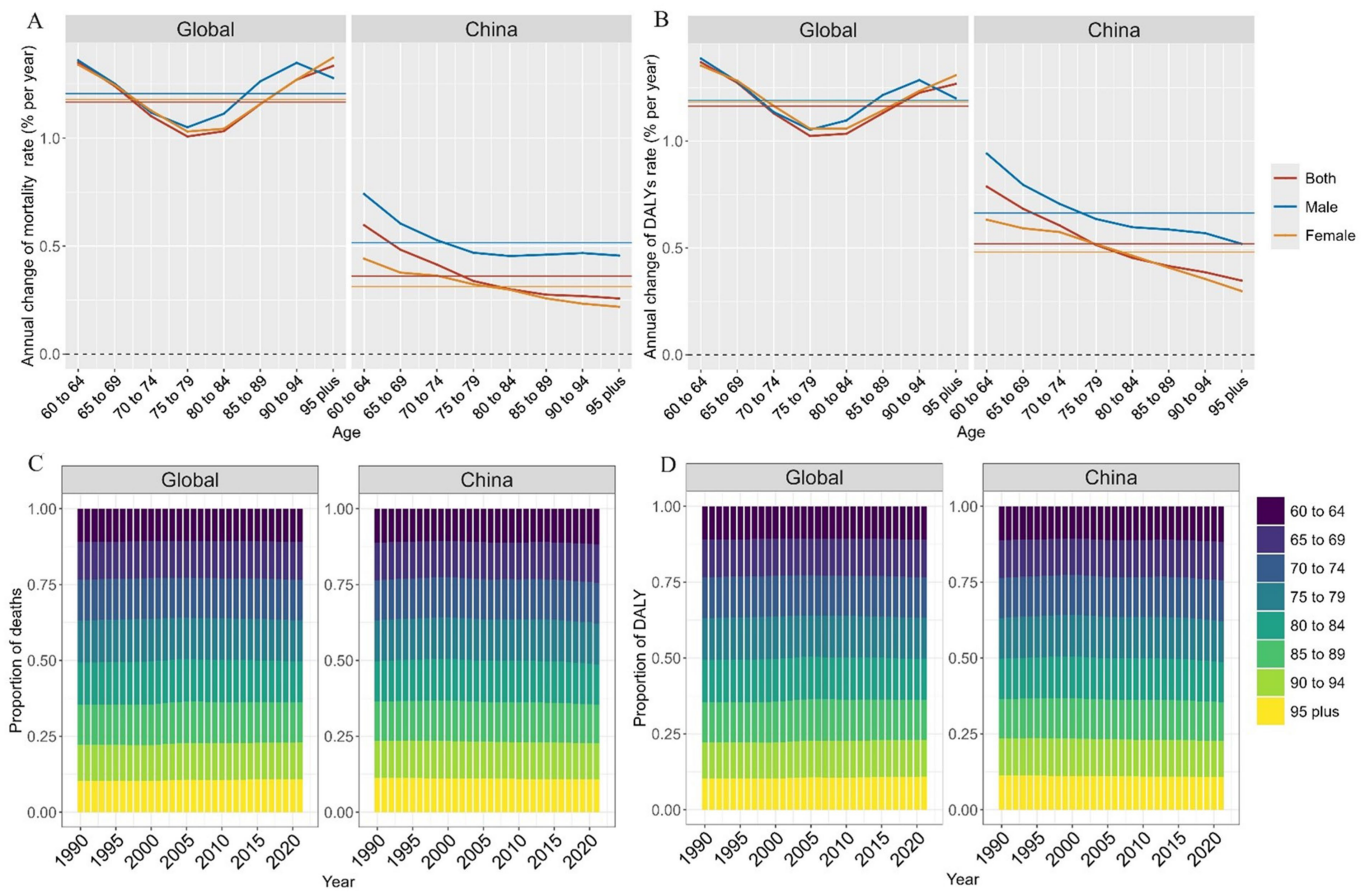
Local drift and age distribution of mortality and DALY rates from 1990 to 2021. DALY, disability-adjusted life year. **(A)** Annual change of mortality rate; **(B)** Annual change of DALYs rate; **(C)** Proportion of deaths; **(D)** Proportion of DALYs.

The annual percentage changes in dementia disease burden attributable to HFPG across different age groups, that is, the local drifts in mortality and DALYs rates derived from the APC model, are presented in Figures 2A,B and Supplementary Tables S3, S4. In China and globally, the mortality and DALYs rates have demonstrated an overall increasing trend across all age groups and sexes. Globally, a U-shaped trend is observed with increasing age. In contrast, in China, the annual percentage increase in mortality and DALYs rates showed a downward trend with increasing age. The annual percentage increase in males was significantly higher than that in females across all age groups. Compared with the global level, the local drift in both mortality and DALYs rates in China is much lower, at less than 1% per year.

Temporal changes in the age distribution of deaths and DALYs attributable to HFPG are illustrated in Figures 2C,D and Supplementary Tables S5, S6. The age distributions of deaths and DALYs in China and globally were similar and the overall trend of change over time was flat. The absolute number of deaths and DALYs were nearly evenly distributed in each age group. Although the >95 years age group did not have the highest number of deaths due to HFPG, the mortality rate was the highest due to the relatively small population in this age group.

### 3.3 Age, period, and birth cohort effects on dementia disease burden attributable to HFPG

**Figure 3:**
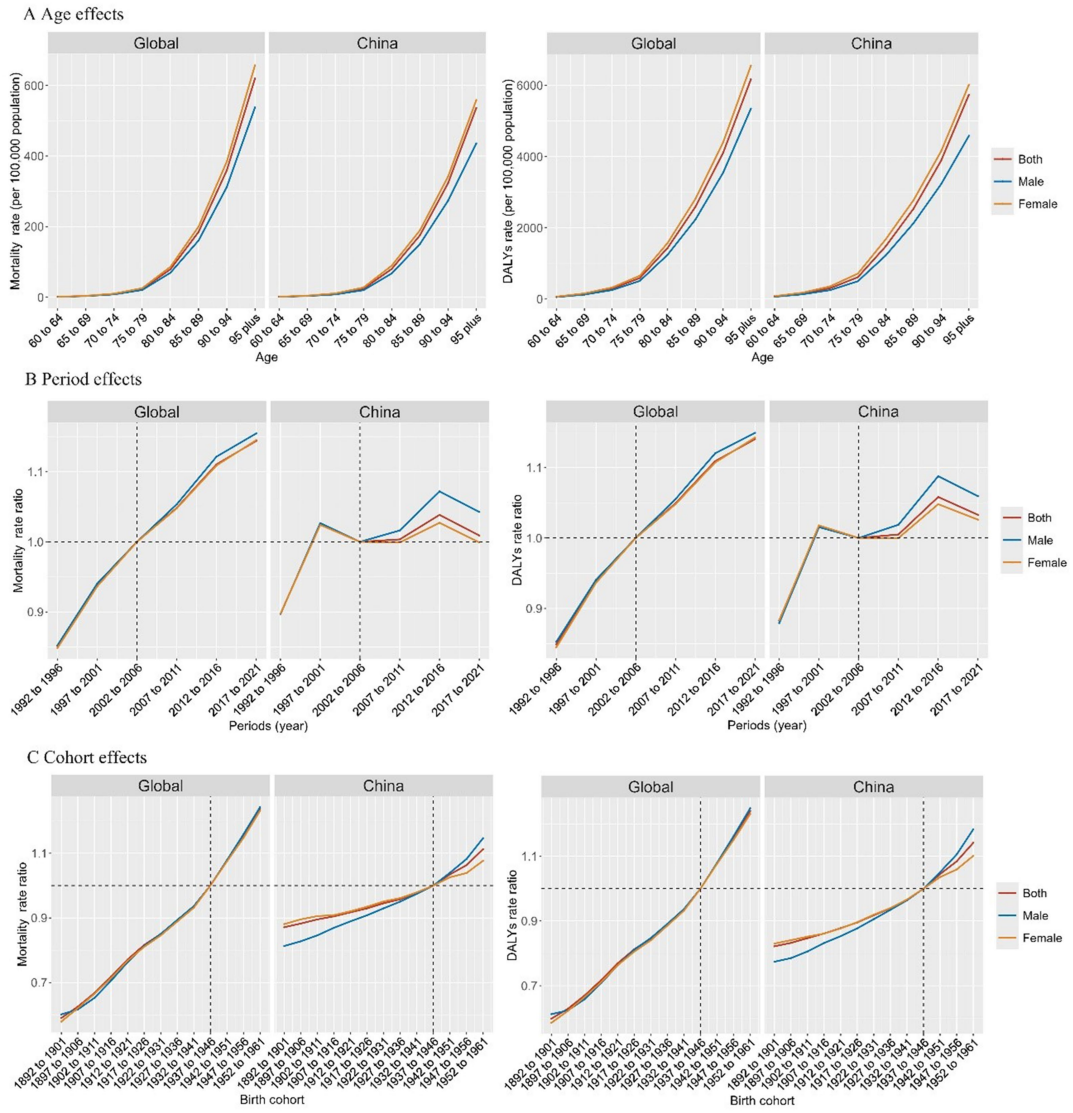
Age, period, and birth cohort effects on burden of dementia attributable to high fasting plasma glucose. DALY, disability-adjusted life year. **(A)** Age effects of mortality and DALYs; **(B)** Period effects of mortality and DALYs; **(C)** Cohort effects of mortality and DALYs.

The age, period, and birth cohort effects of the APC models are shown in Figure 3 and Supplementary Tables S7–S12. Generally, for both mortality and DALYs rates, age effects presented similar patterns in China and globally, with the lowest risk existed in the 60–64 years group with risk increasing with age and reaching the highest in the >95 years age group (Figure 3A). In addition, females had higher mortality and DALYs rates than males across all age groups. Globally, period effects show an overall constantly increasing risk of mortality and DALYs rates. In China, it is worth noting that the period effects presented a declining risk after the 1997–2001 period, which may have been due to the decision to promote health reform and development promulgated in 1997. After 2011, another rising and declining risk pattern was observed. This may be due to the promotion of new healthcare reforms in China in 2009. The first upward risk may be due to the increased screening and diagnosis of hyperglycemia after the new healthcare reform, followed by a decrease in risk due to better management and control of diabetes and dementia (Figure 3B). For birth cohort effects, overall, constantly rising risks of mortality and DALYs rates were observed both in China and globally.

Temporal trends in the burden of dementia attributable to HFPG in several exemplary countries across different SDI quintiles with age, period, and birth cohort effects are shown in Supplementary Figures 2, 3. Increasing trends in mortality and DALYs rates were observed across all age groups in all the exemplary countries. Compared with these exemplary countries, the overall annual percentage increase in China was lower, at less than 1% per year, across all age groups. Additionally, in contrast to China, the annual percentage increase in the USA, a country with high SDI, increased with age. In the APC models, for both mortality and DALYs rates, the age effects increased with age, and the period and cohort risks worsened in recent years in all exemplary countries, whereas the period risks in China showed inflection points of decline.

### 3.4 Forecasted mortality and DALYs rates in 2040

**Figure 4:**
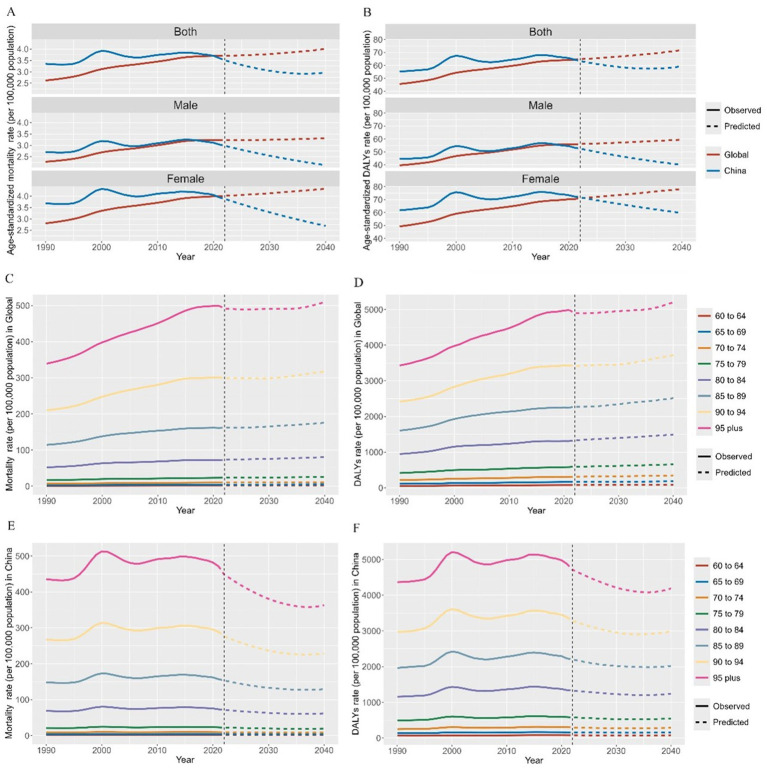
Forecasted mortality and DALY rates of dementia attributable to high fasting plasma glucose in 2040. DALY, disability-adjusted life year. **(A)** Age-standardized mortality rate; **(B)** Age-standardized DALYs rate; **(C)** Mortality rate in Global; **(D)** DALYs rate in Global; **(E)** Mortality rate in China; **(F)** DALYs rate in China.

We demonstrated trends in mortality and DALY rates of dementia attributable to the HFPG from 1990 to 2021 and forecasted future rates in 2040 (Figure 4). By 2021, it is worth noting that there was a gradual downward trend in ASMR (annual percent change = −1.81, *p* < 0.001) and ASDR (annual percent change = −0.92, *p* < 0.001) in China after 2015, while the global trend was consistently upward (ASMR: annual percent change = 0.17, *p* < 0.001; ASDR: annual percent change = 0.37, *p* < 0.001). Similarly, both the forecasted ASMR and ASDR from 2022 to 2040 demonstrate an increasing trend globally and a declining trend in China (Figures 4A,B and Supplementary Tables S13, S15). Notably, in China, the decline in ASMR was significantly higher than that in ASDR. The ASMR is expected to decline by 17.7% from 3.60 in 2021 to 2.97 in 2040, whereas the ASDR is expected to decline by 7.9% from 64.48 in 2021 to 59.38 in 2040. In addition, the increase in males from 1990 to 2021 was higher than that in females; by 2040, the decline in males is projected to be slightly lower than that in females for ASMR (30.0% vs. 31.8%) but higher for ASDR (24.9% vs. 17.5%). Globally, all age groups showed increasing trends for both mortality and DALY rates by 2040 (Figures 4C,D and Supplementary Tables S14, S16). In China, an inverse downward trend was observed in all age groups. The percentage of decline increased with age, reaching the highest in the >95 years group (23.0% for ASMR and 14.1% for ASDR) (Figures 4E,F).

## 4 Discussion

In this study of adults aged >60 years, we found that the dementia disease burden attributable to HFPG was on an upward trend in China and globally from 1990 to 2021. However, China showed a much slower upward trend than the global level, and after 2015, it showed a declining trend. Local drifts in mortality and DALY rates showed a downward trend in China with increasing age. In the APC models, age and birth cohort effects presented a constant rising risk both in China and globally, while period effects showed a constant rising risk globally and two inflection points in China. Additionally, the ASMR and ASDR were higher in middle-to-high SDI countries than in low SDI countries, and higher in females than in males. The forecasted ASMR and ASDR by 2040 demonstrated an increasing trend globally and a declining trend in China. Notably, the decline in ASMR was significantly greater than that in ASDR in China.

HFPG leads to cognitive decline by triggering systemic inflammation, disrupting the blood–brain barrier, and causing hippocampal atrophy (29). Additionally, insulin resistance impairs brain cognitive function and promotes the formation of *β*-amyloid plaques, further exacerbating neurodegeneration (30). Li et al.’s study also confirmed that elevated fasting glucose is a risk factor for dementia (31). The disease burden of dementia attributable to HFPG was highly heterogeneous across regions. Our results demonstrated that the ASMR and ASDR of dementia attributable to HFPG were higher in countries with middle-to-high SDI. These findings reveal that the disease burden of dementia attributable to the HFPG may be heavier in countries with a higher SDI. This could be attributed to several factors. First, life expectancy in high-SDI countries is higher than that at the global level. Second, the case surveillance and reporting systems are better developed in high SDI countries, which enables early recognition and diagnosis of hyperglycemia and dementia (32). Third, due to the progress of urbanization, unhealthy dietary patterns, and lower physical activity levels, the prevalence rates of metabolic diseases (hypertension, type 2 diabetes mellitus, and non-alcoholic fatty liver disease) will show the greatest growth in high SDI countries from 2000 to 2021 (33). These adverse trends in lifestyles and metabolism have contributed to the increased burden of hyperglycemia and subsequent dementia. Therefore, countries with a higher SDI may need to prioritize lifestyle and dietary interventions, such as advocating for reduced consumption of high-sugar and high-fat foods and increased levels of physical activity.

In the APC analysis, the age effects showed an exponentially increasing trend after 60 years of age, reaching the highest value in the >95 years age group. This is consistent with the trend in the overall dementia burden across age groups in China (34). Globally, the annual growth rate peaks in individuals <70 years and >80 years. In contrast, the annual growth rate among Chinese residents tends to decline with increasing age, indicating a rapidly growing dementia burden in the relatively younger older population. This suggests a possible trend toward a younger onset of dementia attributable to HFPG within the Chinese context, indicating the need to focus more on the relatively younger older adults. Globally, the older population continues to bear a significant disease burden.

The ASMR and ASDR in China and globally were similar, but the net drift in China from 1990 to 2021 was much lower than that at the global level, which may be due to the effective management of hyperglycemia and dementia during this period in China. Notably, as the period risk demonstrates, compared with the global RR, China presented two inflection points. The first inflection point may be due to the decision to promote health reform and development promulgated in 1997 in China, which led to a modest reduction in the dementia disease burden attributable to HFPG. First, a rising risk was observed and after 2009, a declining risk was observed. The promotion of a new healthcare reform in 2009 in China has played a crucial role in this change (35). The reform enacted a series of policies to manage blood glucose levels and alleviate the burden of diabetes, particularly in the reform of basic public health services (36, 37).

First, for individuals at high risk of diabetes, free screening, diabetes-related health education, lifestyle counseling, and at least four blood glucose tests per year were provided (38). Second, diabetes management was strengthened by establishing health records, conducting annual examinations and return visits, and urging patients to review and take medicine on time. Studies have shown that the treatment coverage for diabetes patients increased from 30.8 to 36.6% after the medical reform (39). Third, the provision of diabetes drugs in grassroots health centers has been greatly improved, and most health centers can meet the daily drug needs of diabetes patients. Fourth, the quantity and capability of health technicians and primary healthcare institutions have been enhanced, thereby improving the efficiency of primary healthcare services (40). Fifth, China has continuously issued preferential policies on the price of diabetes drugs to reduce drug expenditure. The average blood glucose level was substantially controlled after implementation of the new healthcare policies.

Therefore, although the global HFPG-induced dementia risk consistently increased, China showed a declining trend after 2015. The increase in risk in China during 2009–2014 was probably due to the strengthened screening and diagnosis of diabetes and dementia, which is also evidenced by the incidence curve for China shown in the Supplementary Figure 1. This disparity indicates that diabetes management strategies in China have effectively alleviated the HFPG-related dementia burden, potentially providing a reference pattern for other countries in their efforts to reduce this burden. Low- and middle-income countries can learn from China’s experiences with healthcare reforms to gradually expand the coverage of medical security and enhance the level of protection. By adopting a government-led approach with multiparty participation, it is possible to establish a medical security system suitable for the national conditions of the country, thereby alleviating the economic burden on patients with diabetes and their families.

Our study found that females had higher levels of ASMR and ASDR than males, which is consistent with previous studies (34). This may be explained by several reasons. First, females have a longer life expectancy than males due to their inherent biological differences (41, 42). Second, females are more likely to develop Alzheimer’s disease and other dementias than males, possibly owing to differences in brain structure, function, and development (43). A previous study demonstrated that smoking was the leading risk factor for dementia in males, whereas metabolic risks were the primary contributors in females (44). This suggests that policies should prioritize glucose level screening and management in females to better control the subsequent dementia burden among females. However, we also found that the annual percentage increase for males was higher than that for females from 1990 to 2021, suggesting that males may experience a faster growth rate, which is also a cause of concern. This may be due to the increasing prevalence of smoking, alcohol consumption, and high-fat diets among males, which elevates the risk of HFPG and cardiovascular disease, thereby accelerating the onset of dementia in males (45, 46). Additionally, males may be less inclined to seek medical attention, potentially leading to delayed diagnosis and treatment of HFPG and other risk factors (47). However, with social development and advancements in healthcare, patients who were previously undiagnosed are now being identified, contributing to the increased disease burden observed in males.

As for the forecasted disease burden in 2040, the predicted decline in ASMR is much higher than that in DALY (17.7% vs. 7.9%) in China. Although the mortality rate of dementia attributable to HFPG has reduced, the loss of healthy life-years caused by disability is still large. Therefore, it is critical to focus on the impaired the quality of life caused by disabilities attributed to diabetes. The government should introduce crucial policies to strengthen the screening, management, and treatment of diabetic complications and improve the prognosis of patients with diabetes and dementia, thereby reducing the disability caused by diabetes and improving the quality of life of patients.

APC models are valuable for analyzing health trends but suffer from an identifiability problem due to the linear relationship (Age = period – cohort). Traditional APC models often require arbitrary constraints to estimate the parameters, potentially introducing bias. The Bayesian APC addresses this issue by applying smoothing priors, such as second-order random walk priors, which regularize parameter estimates while preserving trends (48). This method addresses the identifiability issue. Sensitivity analysis showed that the model remained stable even after adjusting the parameters used (24). Additionally, Bayesian offers the advantage of quantifying uncertainty, achieving a more robust interpretation, and making it a more flexible and reliable tool for epidemiological research.

The current study has some limitations. Although the GBD provides high-quality estimates of the global burden of disease, it is based on the population level. GBD data may not fully capture the heterogeneity of disease severity across different populations and over time, as it assumes a constant severity distribution (49). Additionally, in data-scarce regions (e.g., low-income countries), its statistical approximations for incidence/prevalence can introduce uncertainty. If we had access to information at the individual level, the results may be more instructive for public health planning efforts. Since the GBD data only provided DALY and mortality rates for risk factors without corresponding incidence and prevalence data, this study was unable to explore trends and distributions in the incidence and prevalence of dementia. This limitation necessitates large-scale surveys and investigations in the future to explore these aspects.

In summary, this study demonstrated that the dementia disease burden attributable to HFPG showed a declining trend in China in recent years, whereas the global trend consistently increased from 1990 to 2021 in adults aged >60 years. Similarly, the forecasted disease burden by 2040 demonstrated a declining trend in China, with a greater decline in the ASMR. The period effects presented two inflection points in China, probably due to healthcare reform. Additionally, mortality and DALYs rates increased with age, whereas the annual growth rate decreased with age in China. The ASMR and ASDR were higher in middle-to high-SDI countries and in females. These results suggest that targeted policies should be introduced to reduce the burden of diabetes and dementia in females and older adults with a greater focus on improving their quality of life. Additionally, keen attention should be paid to the rapid increase in the number of males and the relatively younger older adults. Furthermore, the gradually decreasing dementia burden in China following healthcare reforms indicates that China’s strategies for preventing and managing diabetes and dementia may offer valuable insights for other regions.

## Data Availability

Publicly available datasets were analyzed in this study. This data can be found at: https://vizhub.healthdata.org/gbd-results/.
