# Supplementary material for: Temporal trends in dementias in older adults attributable to high fasting plasma glucose from 1990 to 2021 and forecasted disease burden in 2040 in China and globally
Source: Front Public Health. 2025 Jun 18;13:1584386. doi: 10.3389/fpubh.2025.1584386 (PMC12213440; doi:10.3389/fpubh.2025.1584386)
Supplement: Supplementary file 1 [file Table_1.doc]

Supplementary information for

Temporal trends in dementias in older adults attributable to high fasting plasma glucose from 1990 to 2021 and forecasted disease burden in 2040 in China and globally


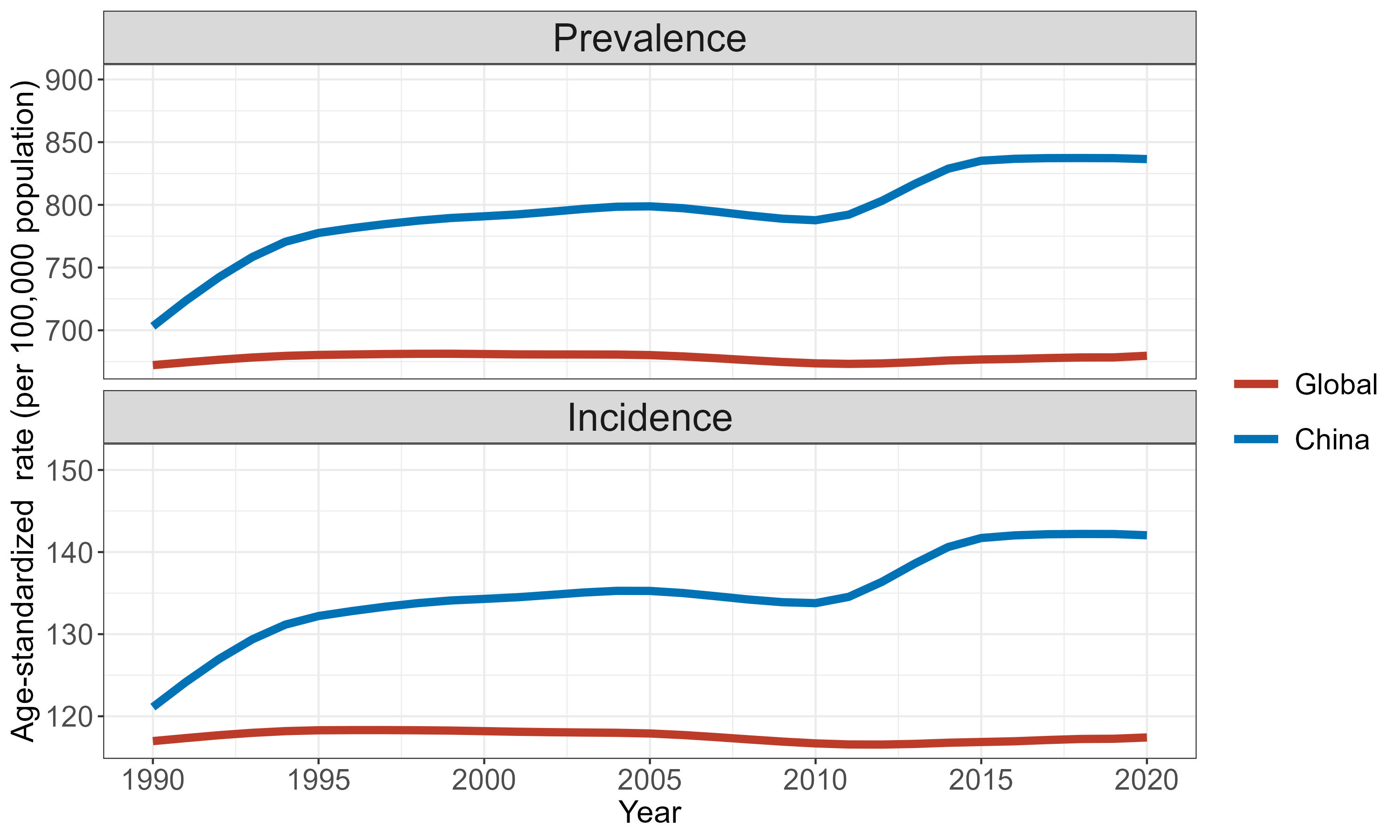


**Figure S1. Prevalence and Incidence of dementias from 1990 to 2021 in Global and China**


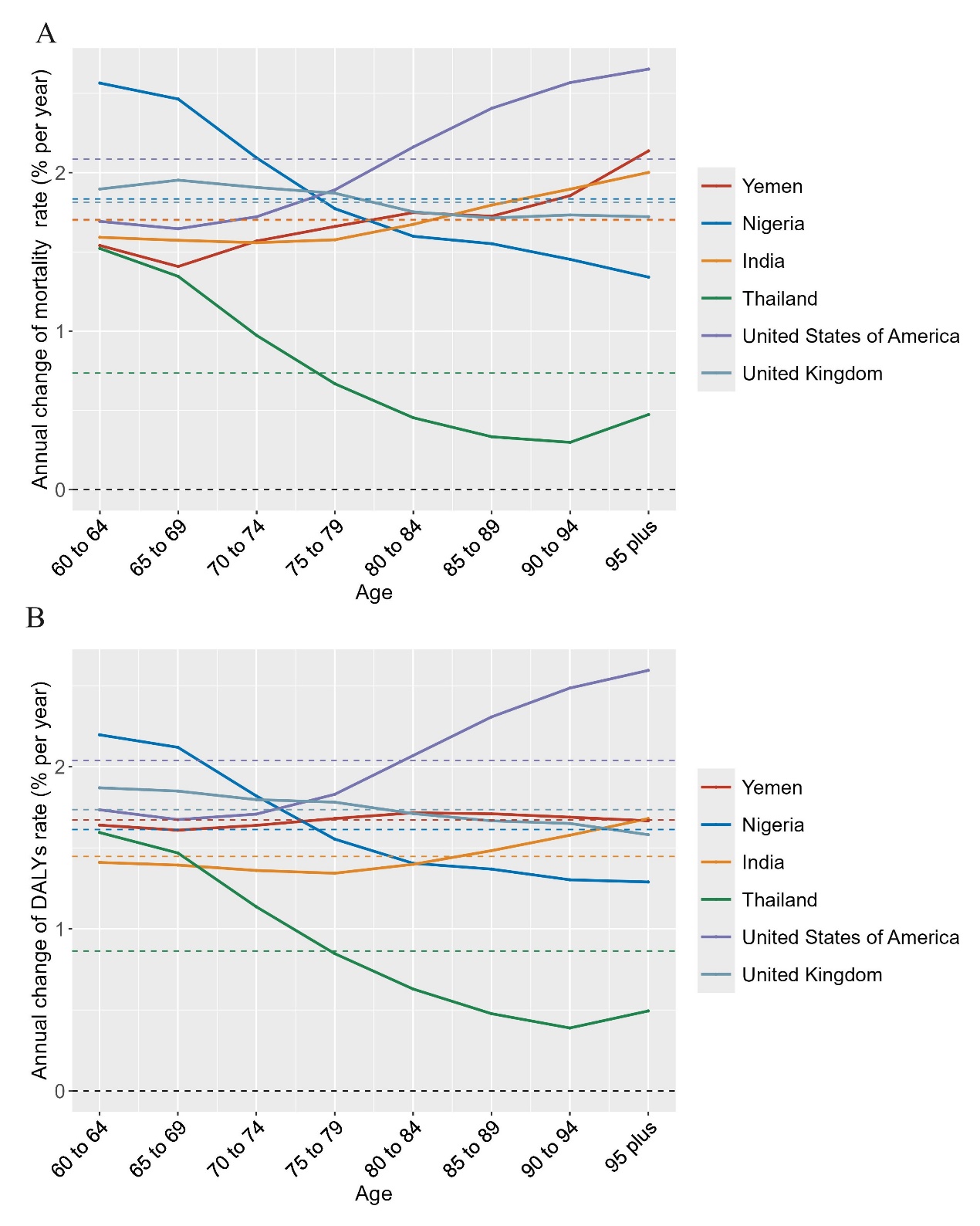


**Figure S2. Local drift and age distribution of mortality and DALYs rates from 1990 to 2021 in exemplary countries**

DALY, Disability-Adjusted Life Years


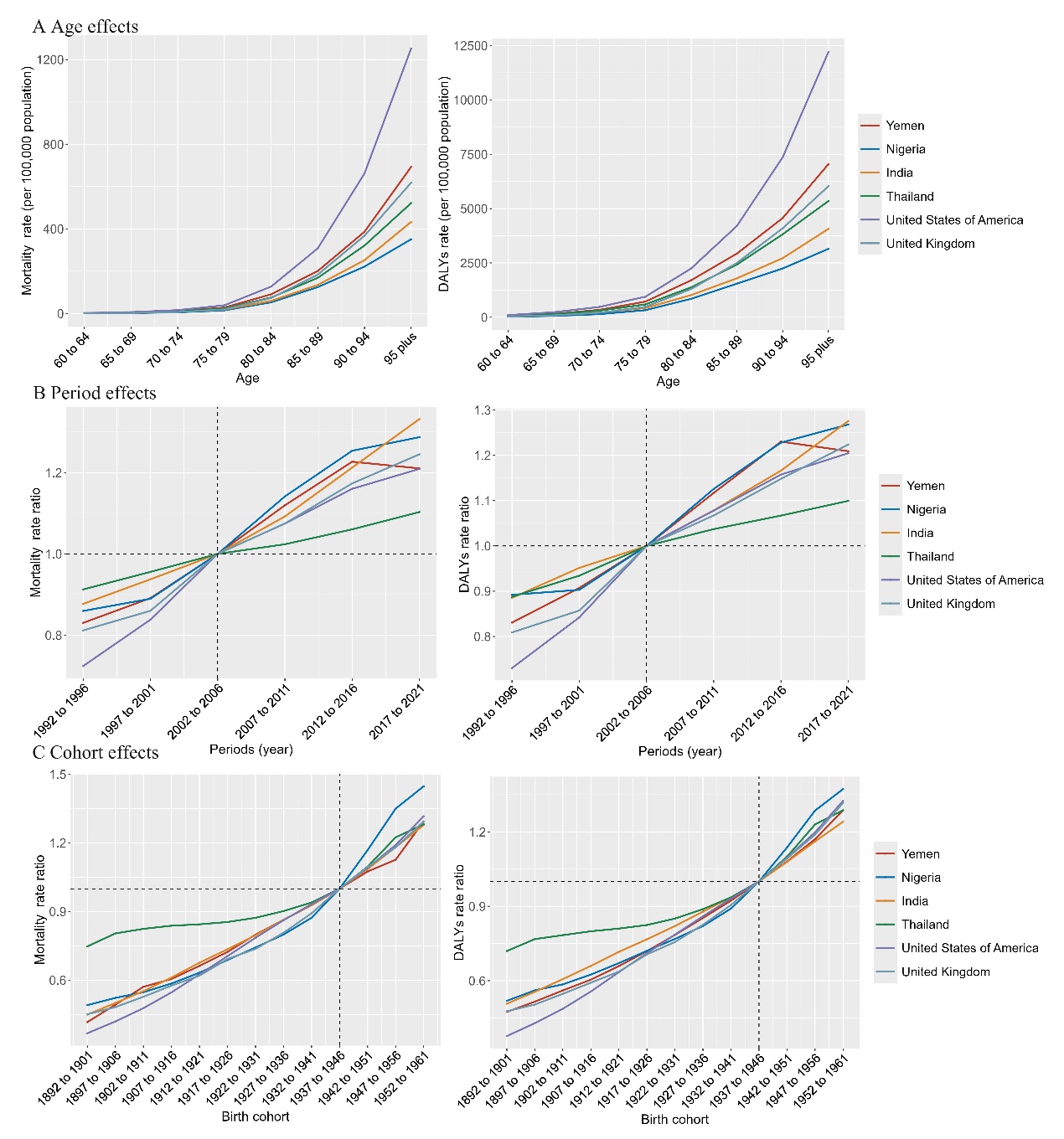


**Figure S3. Age, period and birth cohort effects on burden of dementia attributable to high fasting plasma glucose in exemplary countries**

DALY, Disability-Adjusted Life Years
